# Detecting gas-rich hydrothermal vents in Ngozi Crater Lake using integrated exploration tools

**DOI:** 10.1038/s41598-019-48576-5

**Published:** 2019-08-21

**Authors:** Egbert Jolie

**Affiliations:** 1 GFZ German Research Centre For Geosciences, Telegrafenberg, 14473 Potsdam Germany; 2 ÍSOR Iceland GeoSurvey, Grensásvegur 9, 108 Reykjavík, Iceland; 3 Formerly with BGR Federal Institute for Geosciences and Natural Resources, Stilleweg 2, 30655 Hanover, Germany

**Keywords:** Natural hazards, Hydrology

## Abstract

Gas-rich hydrothermal vents in crater lakes might pose an acute danger to people living nearby due to the risk of limnic eruptions as a result of gas accumulation in the water column. This phenomenon has been reported from several incidents, e.g., the catastrophic Lake Nyos limnic eruption. CO_2_ accumulation has been determined from a variety of lakes worldwide, which does not always evolve in the same way as in Lake Nyos and consequently requires a site-specific hazard assessment. This paper discusses the current state of Lake Ngozi in Tanzania and presents an efficient approach how major gas-rich hydrothermal feed zones can be identified based on a multi-disciplinary concept. The concept combines bathymetry, thermal mapping of the lake floor and gas emission studies on the water surface. The approach is fully transferable to other volcanic lakes, and results will help to identify high-risk areas and develop suitable monitoring and risk mitigation measures. Due to the absence of a chemical and thermal stratification of Lake Ngozi the risk of limnic eruptions is rather unlikely at present, but an adapted monitoring concept is strongly advised as sudden CO_2_ input into the lake could occur as a result of changes in the magmatic system.

## Introduction

Intense gas emissions at the geosphere-atmosphere interface is a common process in volcanically and tectonically active regions. Emission rates of CO_2_ can reach levels of several thousand grams per square meter and day^1^. Due to immediate dilution in the atmosphere continuous CO_2_ emissions do not necessarily result in an acute hazard, unless accumulating in depressions; however, if gas-rich hydrothermal vents are located underwater the risk of limnic eruptions and their disastrous effects as reported for example from Lake Monoun^2^ and Nyos^3–5^ (Cameroon) need to be considered. Herein, the results from a study of Lake Ngozi in the East African Rift System (EARS) are presented, which belongs to the CO_2_-richest lakes worldwide according to a global study^6^.

Ngozi volcano is part of the Rungwe Volcanic Province (RVP), which is located at the triple junction, where the western and eastern branch of the EARS connect^7,8^. The two dominating structural trends in the RVP are NW-SE and NNE-SSW^9^. Lake Ngozi (maximum depth 83 m) is located within the Ngozi caldera and its maximum extent is 2.6 km (W-E) by 1.6 km (N-S). The annual average of the surface water temperature (undefined depth) is 21 °C with temperature variations from 20.0 °C in August to 21.5 °C in March/October^10^. The high salinity and gas content of the lake is explained by assumed fault-controlled inflows of gas-rich, mantle-derived fluids^10^; however, the existence of such structures could not be proved so far. Geochemical analyses^10^ of Lake Ngozis water column do neither indicate a chemical nor thermal stratification as described for Lake Nyos and Monoun^11^. High Na-Cl and low Mg concentrations suggest an inflow of a mature geothermal fluid into the lake, combined with CO_2_ input from regional deep degassing sources and H_2_S, indicated by high pCO_2_ and medium SO_4_ content^10^, respectively. In this way, the system is heated from below and does not favor the development of a distinct thermal stratification. The pH-values range from 6.4–6.9. Lower pH, as a result of H_2_S oxidation to SO_4_, is unlikely due to small quantities of inflowing magmatic fluids in relation to the large water body. In comparison to other volcanic lakes Ngozi does not belong to the deep lakes, such as Lake Nyos (210 m), Kivu (485 m) or Albano (167 m), which means that a stratification is less probable. Existing chemical data and the size of Lake Ngozi suggest that the absence of a stratification is stable. Therefore, limited risk of limnic eruptions is assumed, though monitoring measures are advised as sudden CO_2_ recharge^12^ due to renewed or increased gas input cannot be excluded. In that context Lake Ngozi is rather comparable to the geothermally heated Kelud crater lake (Indonesia)^13^ or volcanic lakes in El Salvador^14^, than Lake Nyos, Lake Kivu or Lake Monoun.

Analyses of the steep crater wall gave evidence of a lake breaching event of the southern caldera rim^9^. It is assumed that the Ngozi tuff eruption (<1 ka) built up the southern caldera rim, which was then breached by the newly formed crater lake. This could still be a potential risk of flooding downstream in particular if lake water is suddenly drained as a result of activated instabilities in the crater wall. On the other hand, rapid CO_2_ recharge from below could result in catastrophic CO_2_ output, which might also facilitate lake breaching events. One has to remember, that Ngozi volcano is an active volcano with the last eruption <1 ka ago, and future volcanic activity in the RVP is very likely. The area-wide effects of catastrophic gas bursts have been analysed and demonstrated by numerical modeling^15^.

## Methods

A 150 kHz echo sounder with a depth range from 0.7–100 m was used for the bathymetric survey. Multiple traverses have been performed using an inflatable boat with an electric motor. Depth information was recorded every 10 m at 4,681 sites. Temperature and electrical conductivity (EC) measurements have been performed with a WTW ProfiLine Multi 197i and a 100 m T-EC electrode (accuracy for temperature ±0.2 °C, for EC ±0.5% of value). 31 T-EC-logs have been performed at regularily distributed sites with data readings every 2 m. In 24 locations the sensor was lowered down to the lake floor to obtain ground temperatures. When the lake floor was touched a distinct change in EC was observed. Results from previous studies^10^ have been used for comparison. CO_2_ flux measurements have been performed according to the accumulation chamber technique^16^ by a floating flux chamber (West Systems). The fluxmeter is equipped with a LI-COR 820 CO_2_ gas analyzer (accuracy <3% of reading). 39 measurements have been conducted on the lake surface, and four reference measurements have been performed on the flank of the crater. All techniques have the great advantage of *in-situ* data readings, which ensure enough flexibility during field campaigns to trace and delineate the main target areas. Data processing was performed with ESRI ArcMap, OriginPro, and FluxRevision software. Data are accessible through the Research Data Repository of GFZ Data Services (dataservices.gfz-potsdam.de) or may be obtained from the author upon request.

## Results and Discussion

**Figure 1:**
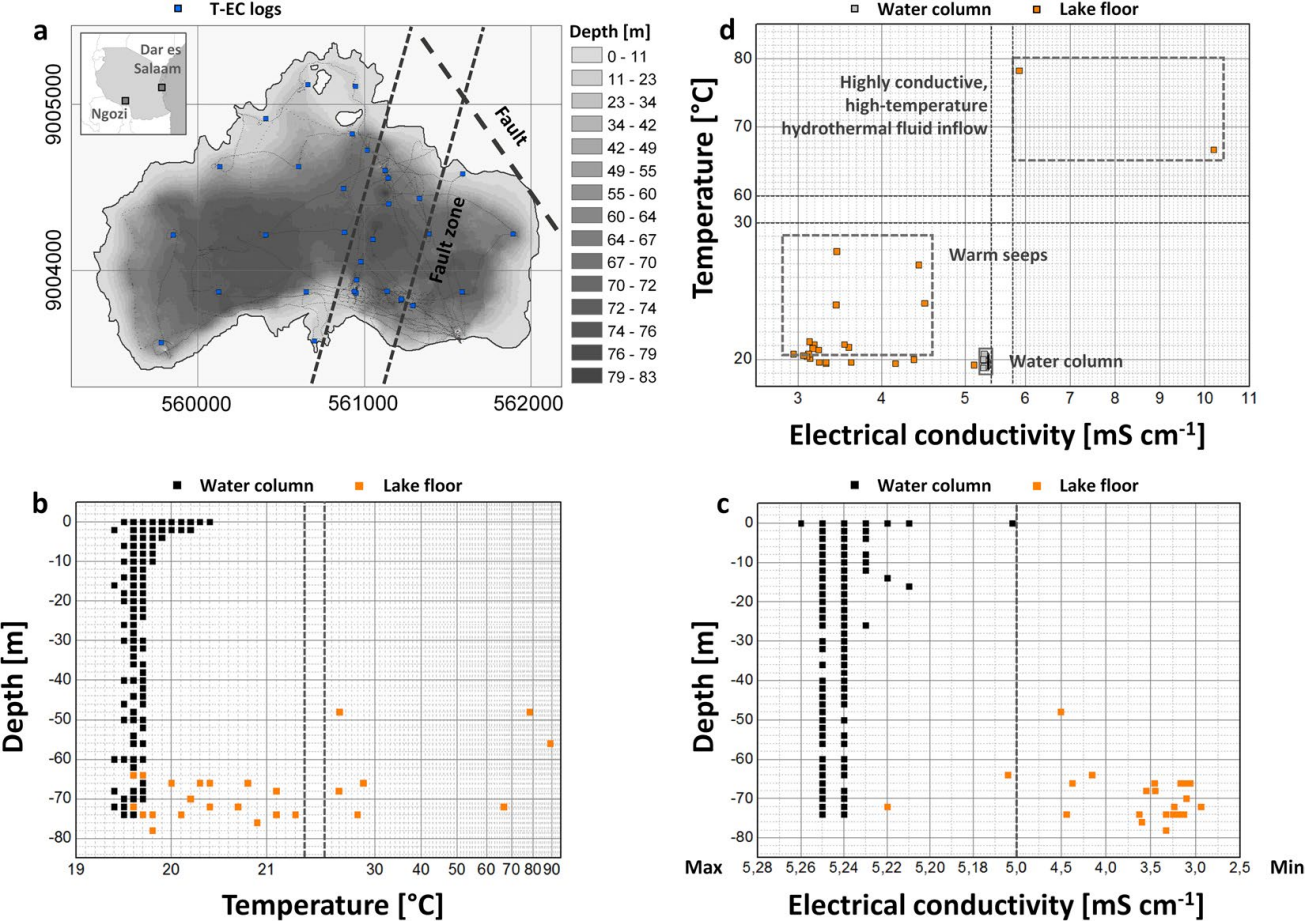
(**a**) Bathymetry of Lake Ngozi obtained from echo sounding (black dots) and locations of T-EC-logs from 31 soundings with 391 water column measurements and 24 lake bottom measurements. A NW-oriented fault^9^ and an inferred NNE-oriented fault zone across the lake floor are illustrated. Tanzania map was obtained from the GinkgoMaps project (www.ginkgomaps.com) and modified for the purpose of this figure. (**b**) Depth vs. temperature plot. (**c**) Depth vs. Electrical conductivity plot. Note that the two highest EC-values (10.2 mS cm^−1^ and 5.9 mS cm^−1^) are not illustrated. (**d**) Electrical conductivity vs. temperature plot. Hydrothermal fluids seem to cause the high electrical conductivity in the lake water. No electrical conductivity reading for data point T = 89 °C. Note the changing scales on x- and y-axes in Fig. 2b–d, which are marked by dashed lines.

Lake Ngozi has a maximum depth of 83 m (Fig. 1a). The western sector is characterized by a relatively flat lake floor, whereas the eastern sector appears rugged. Between both sectors four hole-like structures were identified. The mapped holes are clearly NNE-aligned and suggest the presence of a permeable fault zone, which was not known before. The holes have a maximum depth of 10 m compared to the relatively flat surrounding lake floor.

Lake Ngozi is characterized by very stable temperature conditions in the water column below 10 m depth, where surface effects can be excluded (Fig. 1b). Average water temperature from 10 m depth down to the lake floor is 19.6 ± 0.1 °C. In the uppermost level of the lake, water temperature varies from 20.4 °C at the surface to 19.4 °C at 10 m depth. No significant spatial temperature variations have been observed in different depth levels of the water column. In contrast to the water column, lake floor temperatures show substantial variations from 19.6 °C to 89 °C, detected in the SE and NE of the lake. The same characteristic was observed for electrical conductivity (Fig. 1c). EC variations along the water column were minimal with an average value of 5.25 mS cm^−1^, but EC values of the lake floor (presumably in sediments) varied substantially from lower to higher values (see also Kusakabe *et al*., 2019)^17^ (min. 3.1 mS cm^−1^; max. 10.2 mS cm^−1^). The average electrical conductivity of Lake Ngozi indicates the presence of large quantities of dissolved ionic components, which can only be the result of major subaquatic hydrothermal feed zones. The highest EC values have been measured at sites characterized by highest ground temperatures (Fig. 1d), possibly indicating inflow of hydrothermal fluids through the lake floor.

**Figure 2:**
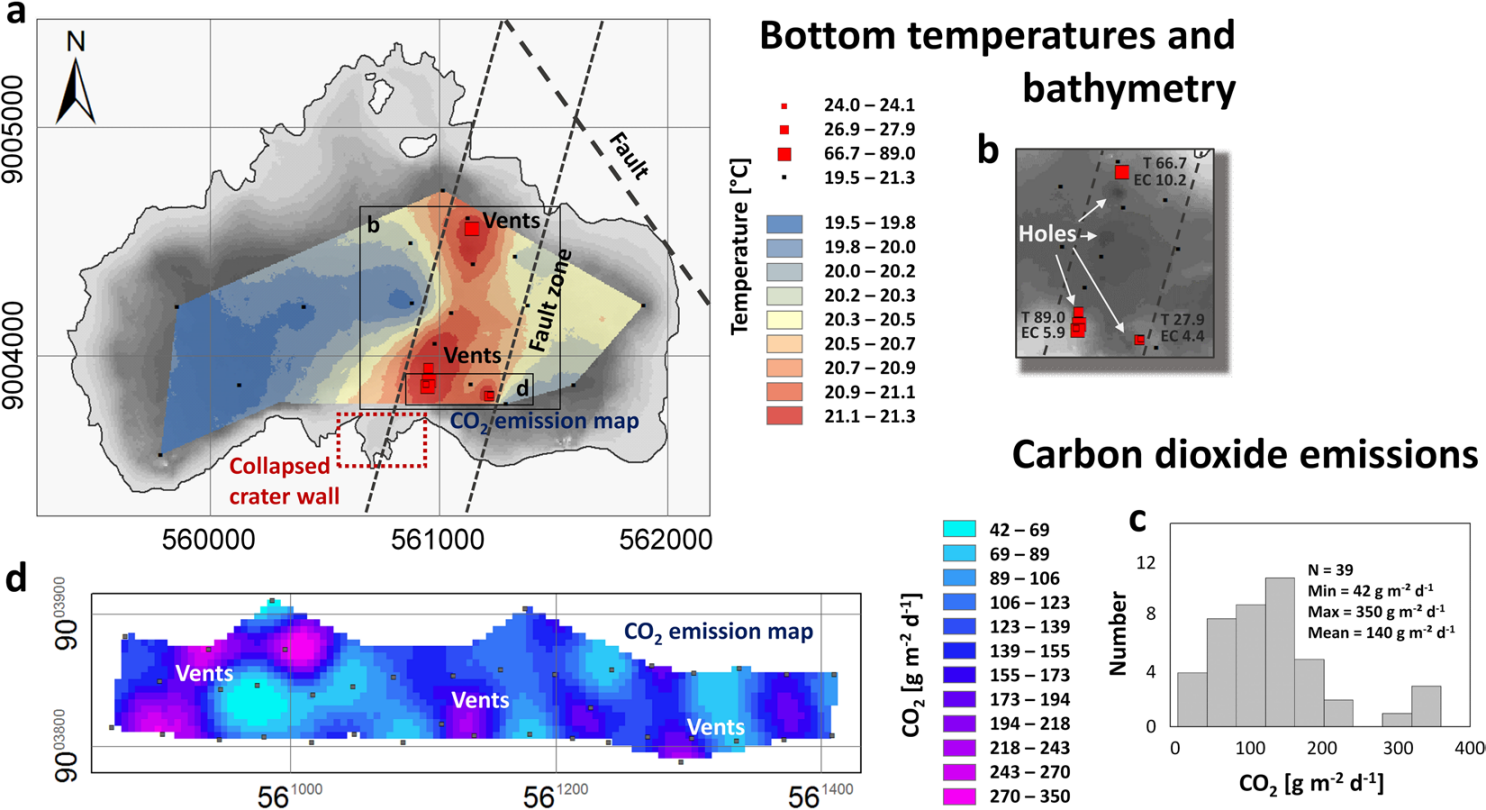
(**a**) Thermal map of lake floor. The red box indicates the location of the collapse structure in the southern crater wall. (**b**) Identified hole-like structures in the central part of the lake with ground temperatures and electrical conductivity values. Maximum temperatures T [°C] and electrical conductivities EC [mS cm^−1^] along the central corridor are illustrated. (**c**) Histogramm of CO_2_ flux measured on the lake surface. (**d**) Gas flux measurements were undertaken at the water surface of Lake Ngozi (2,064 m asl) by means of a floating accumulation chamber. Two profiles have been measured (39 measurements on lake + 4 control measurements onshore) across an area with known thermal anomalies on the lake floor. Major gas emissions have been observed by gas bubbling at the water surface and smell of H_2_S. CO_2_ flux varied between 42–350 g m^−2^ d^−1^ with a mean value of 140 g m^−2^ d^−1^. Maximum CO_2_ emissions were detected across the major thermal anomaly.

With the purpose to identify minor ground temperature variations a temperature map was compiled, excluding all temperature values above 21.3 °C (Fig. 2a). The map illustrates a well-defined thermal anomaly along a central NNE-oriented corridor, which can even be identified with low temperature variations of less than d1.8 K and without any information of high ground temperatures. Ground temperatures above 21.3 °C (up to 89 °C) are illustrated as points and plot in the same area, thus confirming the pattern of the temperature map. It is expected that further high-temperature spots are located in the deep holes identified along the NNE-oriented corridor (Fig. 2b).

CO_2_ emissions from Lake Ngozis water surface have been determined across an area with increased lake bottom temperatures and identified hole structures (Fig. 2c,d) in order to link gas flux with permeable fractures in the subsurface^18^. Gas flux ranges from 42–350 g m^−2^ d^−1^ with an average of 140 g m^−2^ d^−1^, comparable to gas emissions from other volcanic lakes^19^. Reference measurements on the flank of the crater range from 2–16 g m^−2^ d^−1^ with an average of 9 g m^−2^ d^−1^.

## Conclusion

It is demonstrated how basic field studies can significantly improve the understanding of potential subaquatic hydrothermal feed zones in volcanic lakes. One of the striking results of the study is that minor variations in lake floor temperatures between 19.5 °C and 21.3 °C (d1.8 K) already pinpoint thermal anomalies along a narrow, NNE-oriented corridor, which suggests a strong fault control. Deep holes identified by an echo sounding survey seem to be the centres of the thermal anomalies, where gas-rich fluids enter the lake. This was confirmed by increased gas emissions from the lake surface. Increased lake floor temperatures correlate with increased electrical conductivity, suggesting intense fluid-rock interaction and possible formation of alteration minerals. The success of the presented study is based on the combination of different methodological approaches. The site-specific findings are also meaningful for a conceptual advance in the understanding of the Ngozi hydrothermal system and related geothermal exploration activities, as upflowing hydrothermal fluids have always been suspected under Ngozi volcano, but could never be proved before this study.

Lake Ngozi seems to be mainly affected by a thermally driven mixing^20^. Weak convective mixing as a result of fluid inflow from the geothermal system cannot be seen in the existing data, but also cannot be excluded due to possible variations in the magmatic-geothermal system underneath. The presented data can build the basis for the development of a site-specific monitoring concept for the main areas with increased gas flux and ground temperatures. The main hazard at Lake Ngozi is the reactivation of the magmatic system, which would have severe implications on the degassing processes. The permanent installation of a differential absorption LIDAR (DIAL) system^21^ in combination with thermal sensors could be a suitable solution to detect precursory signals of volcanic unrest. Further methodological improvements can be achieved by thermal mapping of the lake floor using fibre optic measurements for integrated temperature logs, and direct sampling of hydrothermal fluids at the identified feed zones.
